# Ability of Linezolid to Combat *Staphylococcus aureus* and *Pseudomonas aeruginosa* Isolated from Polymicrobial Wound Infections

**DOI:** 10.3390/antibiotics14060597

**Published:** 2025-06-11

**Authors:** Samar A. Ahmed, Vy T. Luu, Teresa C. Oyono Nsuga, Steven E. Burgos, Eugene Kreys, Jered Arquiette, Justin R. Lenhard

**Affiliations:** 1 Department of Clinical and Administrative Sciences, California Northstate University College of Pharmacy, Elk Grove, CA 95757, USA; samar.ahmed@cnsu.edu (S.A.A.); vy.tranluu4045@cnsu.edu (V.T.L.); teresa.oyononsuga8800@cnsu.edu (T.C.O.N.); steven.burgos5252@cnsu.edu (S.E.B.);; 2 Department of Pharmacy, San Joaquin General Hospital, French Camp, CA 95231, USA

**Keywords:** linezolid, *Staphylococcus aureus*, *Pseudomonas aeruingosa*, pharmacodynamics, polymicrobial, clindamycin, host defense peptides, wound infections

## Abstract

**Background/Objectives**: The optimal therapy for polymicrobial wound infections is poorly defined. We sought to characterize the ability of linezolid to combat mixed cultures of *Staphylococcus aureus* and *Pseudomonas aeruginosa*. **Methods**: The antistaphylococcal activity of linezolid was assessed in 24-h time-killing experiments that used *S. aureus* and *P. aeruginosa* isolated from polymicrobial wound infections. Clindamycin was also evaluated as a comparator. A Hill-type mathematical model was used to assess the maximum killing of *S. aureus* (E_max_). The ability of linezolid to potentiate the activity of host defense peptides against *P. aeruginosa* was evaluated using LL-37. **Results**: In the presence of *P. aeruginosa*, the E_max_ of linezolid decreased in 5/9 co-culture experiments and increased in 4/9 co-culture experiments in comparison to linezolid against *S. aureus* alone. The potency of linezolid was not significantly impacted by the presence of *P. aeruginosa*. In comparison, the maximal *S. aureus* killing achieved by clindamycin decreased in eight out of nine experiments, and somewhat paradoxically, the potency increased in nine out of nine experiments. In the host defense peptide assay, the supratherapeutic linezolid concentration of 64 mg/L did not significantly enhance the killing of the LL-37 peptides (*p* ≥ 0.121), but the concentration of linezolid was significantly associated with the killing of one of three *P. aeruginosa* isolates (*p* = 0.005). **Conclusions**: *P. aeruginosa* had a minimal impact on the antistaphylococcal activity of linezolid in comparison to clindamycin. Linezolid did not exert a consistent ability to enhance the antipseudomonal activity of host defense peptides. These data may help inform antimicrobial selection during polymicrobial wound infections.

## 1. Introduction

*Staphylococcus aureus* and *Pseudomonas aeruginosa* are two of the most notorious pathogens in the modern healthcare system [1]. Both organisms are responsible for infections at many sites in the body, but in wound infections specifically, *S. aureus* and *P. aeruginosa* represent the two pathogens most commonly encountered in the clinic [2]. Not only are both organisms isolated separately during the treatment of wound infections, but *S. aureus* and *P. aeruginosa* are commonly cultured from the same infected wound [3]. Co-infection may enhance bacterial virulence, treatment resistance, and worsen clinical outcomes, potentially due to interspecies competition that promotes the upregulation of exoproducts, surface proteins, and biofilm formation [4]. Given the frequency of encountering both organisms, it is important to understand how to combat both pathogens when they are simultaneously cultured from a single wound infection.

One of the most noteworthy phenomena to consider when combatting polymicrobial *S. aureus* and *P. aeruginosa* infections is the altered susceptibility of both pathogens to clinically relevant antimicrobial agents. Although some investigations suggest that *S. aureus* may alter the susceptibility of *P. aeruginosa* to certain drugs [5,6], the most common observation is that *P. aeruginosa* alters the susceptibility of *S. aureus* to common anti-staphylococcal drugs. Depending on the class of antimicrobials and the specific exoproducts released by a strain of *P.* aeruginosa, the ability of antibacterial drugs to kill *S. aureus* may be increased or attenuated [5,6,7,8,9,10,11,12,13,14]. One generalization across multiple studies is that the anti-staphylococcal activity of protein synthesis inhibitors such as aminoglycosides, tetracyclines, and clindamycin is typically decreased by *P. aeruginosa* [10,11,12,14].

Linezolid is an oxzolidinone protein synthesis inhibitor antibacterial agent recommended by the Infectious Diseases Society of America as an option for the treatment of skin and soft tissue infections [15,16]. A recent meta-analysis found that linezolid has a comparable safety profile to vancomycin and may even result in superior outcomes when combatting serious MRSA infections [17]; however, the ability of the drug to combat *S. aureus* in polymicrobial wound infections with *P. aeruginosa* is poorly defined. In a clinical trial that evaluated the use of linezolid to treat diabetic foot infections, patients that cultured *P. aeruginosa* and patients that cultured *S. aureus* had similar clinical cure rates despite the intrinsic resistance of *P. aeruginosa* to linezolid [18]. One possible hypothesis for the comparable outcomes between the two patient groups may be that linezolid increased the clearance of the *P. aeruginosa* by the host immune system, but to the best of our knowledge, such a hypothetical claim is not supported by any studies in the literature.

Host defense peptides represent one of the main countermeasures utilized by the innate immune system against *P. aeruginosa* [19]. In mammals, the most common defense peptides are defensins and cathelicidins, but in humans, the only known cathelicidin is LL-37. Not only is LL-37 used extensively by neutrophils, but epithelial cells, monocytes, lymphocytes, and other cells use the peptide as well. LL-37 has demonstrated considerable activity against *P. aeruginosa* in vitro [20,21,22,23,24], and studies of patients with bronchiectasis suggest that LL-37 levels in sputum may be higher during pseudomonal infections in comparison to infections caused by other pathogens [21,25]. Previous studies have evaluated the potential synergy between antipseudomonal drugs and LL-37 [24,26]. Other investigations observed that antibacterial drugs that were individually inactive against certain bacterial pathogens were capable of enhancing the LL-37 mediated clearance of the bacteria [27,28], but to the best of our knowledge, it is unknown if linezolid may potentiate the activity of LL-37 against *P. aeruginosa.*

In the current study, we sought to determine if linezolid is able to retain its ability to kill *S. aureus* in the presence of *P. aeruginosa*. In vitro time-killing experiments were conducted against two pairs of *S. aureus* and *P. aeruginosa* isolates cultured from the same wound infections. Clindamycin was used as a comparator to represent the level of anti-staphylococcal attenuation exerted by *P. aeruginosa* against a different protein synthesis inhibitor. Finally, the ability of linezolid to increase the host defense peptide-mediated clearance of *P. aeruginosa* was assessed to better define the utility of linezolid during polymicrobial infections. We believe this is one of the first studies to compare the ability of an oxazolidinone versus another protein synthesis inhibitor to combat Gram-positive and Gram-negative polymicrobial communities. This study also provides novel insight into the capability of linezolid to activate a component of the immune system to help eliminate Gram-negative pathogens.

## 2. Results

### 2.1. Time-Killing Experiments

**Figure 1:**
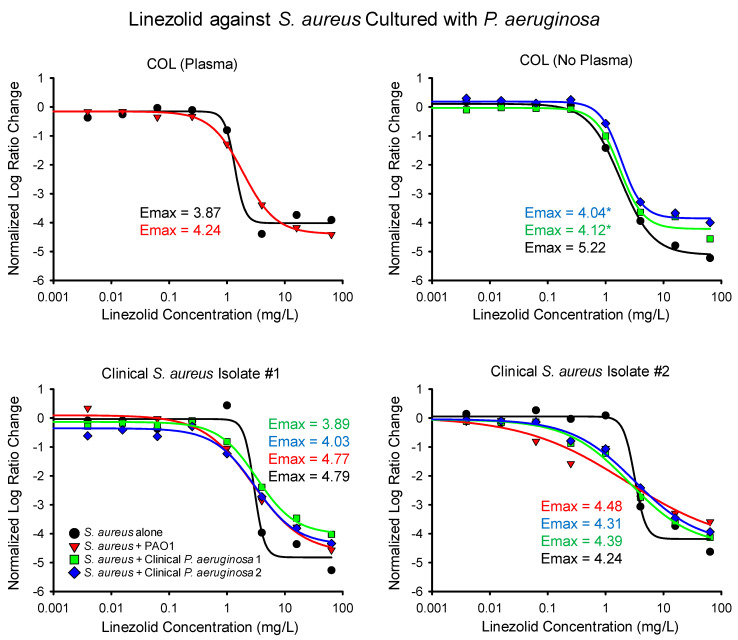
The normalized Log Ratio Changes of *S. aureus* exposed to linezolid are depicted for *S. aureus* alone and cultured with each of the three *P. aeruginosa* isolates. Data points represent averaged values from replicated runs. The *S. aureus* strain COL was grown with bovine plasma when cultured with PAO1, as described in the methodology. The maximum activity of linezolid (E_max_) is listed next to each experiment and statistically significant differences from the corresponding monoculture experiment are noted with an asterisk.

The results of the linezolid time-killing experiments are summarized numerically in Table 1 and graphically in Figure 1. In comparison to monoculture experiments, the E_max_ of linezolid decreased during five out of nine co-culture experiments and increased in four out of nine experiments, respectively. When the *S. aureus* strain COL was exposed to linezolid while grown in bovine plasma, the maximal killing during the monoculture and co-culture differed by less than 10%. Similarly, when the second clinical isolate of *S. aureus* was incubated with linezolid, the maximal activity of the drug differed by less than 6% during the monoculture and co-culture with each of the *P. aeruginosa* isolates. Against the first clinical *S. aureus* isolate, the E_max_ of linezolid, decreased by about 19% during the co-culture with the first *P. aeruginosa* clinical isolate, but the difference was not statistically significant.

The only significant change in the maximal killing of linezolid against the *S. aureus* was observed when COL was cultured with the two clinical isolates of *P. aeruginosa*. During co-culture of COL with the first clinical *P. aeruginosa* isolate, the normalized E_max_ of linezolid decreased from 5.22 in monoculture down to 4.12, a decrease of approximately 20%. The activity of linezolid was also blunted when COL was cultured with the second clinical *P. aeruginosa* isolate, resulting in an E_max_ of 4.04 or a 23% reduction in activity. It is not clear if the lack of bovine plasma may partially explain why a larger shift in the linezolid E_max_ was observed when COL was cultured with the clinical *P. aeruginosa* isolates in comparison to PAO1.

**Figure 2:**
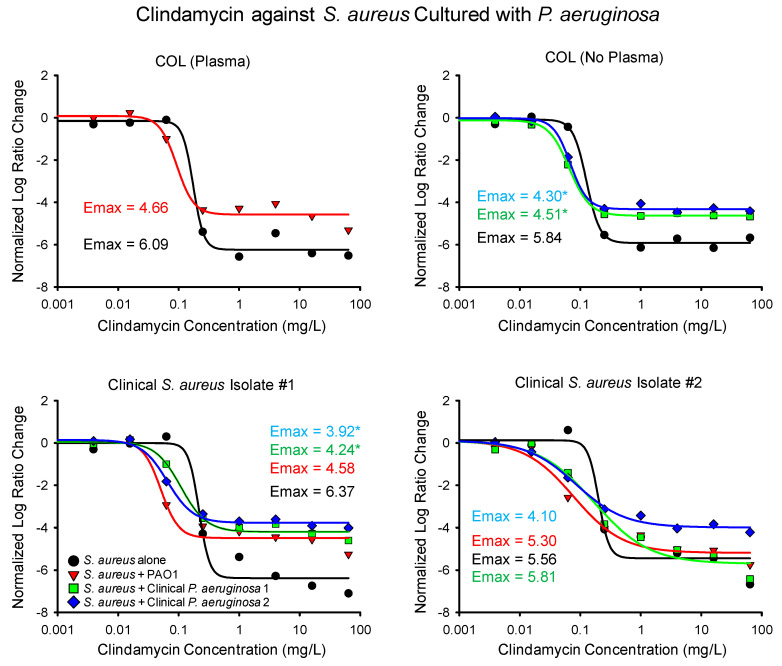
The normalized Log Ratio Changes of *S. aureus* exposed to clindamycin are depicted for *S. aureus* alone and cultured with each of the three *P. aeruginosa* isolates. Data points represent averaged values from replicated runs. The *S. aureus* strain COL was grown with bovine plasma when cultured with PAO1, as described in the methodology. The maximum activity of linezolid (E_max_) is listed next to each experiment and statistically significant differences from the corresponding monoculture experiment are noted with an asterisk.

In comparison to linezolid, exposure to clindamycin resulted in a more consistent reduction in the maximal activity of the drug, as shown in Figure 2 and Table 1. During exposure to clindamycin, the presence of *P. aeruginosa* resulted in a reduction in the clindamycin E_max_ in eight out of nine co-culture experiments. The only observed increase in the clindamycin E_max_ occurred when the second clinical *S. aureus* isolate was cultured with the first *P. aeruginosa* isolate, which resulted in a non-significant increase in the clindamycin E_max_ from 5.56 to 5.81 (~4%).

The most pronounced decreases in the maximal killing of clindamycin were observed for COL and the first clinical *S. aureus* isolate. When COL was cultured with the clinical *P. aeruginosa* isolates, the clindamycin E_max_ significantly decreased from 5.84 to 4.30 and 4.51, respectively. The E_max_ of clindamycin observably decreased when COL was cultured with PAO1, though the ~23% decrease did not reach the threshold for statistical significance in the Log Ratio Change analysis. The maximal killing of clindamycin was also significantly attenuated when the first *S. aureus* clinical isolate was cultured with both clinical *P. aeruginosa* isolates, leading to significant decreases in the clindamycin E_max_ from 6.37 down to 4.24 and 3.92, respectively. During the co-culture of the first *S. aureus* isolate and PAO1, the E_max_ of clindamycin decreased by ~28%, but the change did not meet the threshold of statistical significance.

Unlike the observed decrease in the maximal activity of clindamycin during co-culture experiments, the presence of *P. aeruginosa* actually increased the potency of clindamycin. In all nine co-culture experiments, the concentration of clindamycin needed to achieve half the maximal killing of the drug (EC_50_) was lower when *S. aureus* was grown with *P. aeruginosa* (Table 1), which can be observed as a left shift in the inflection of the sigmoidal curves in Figure 2. Four of the increases in clindamycin potency were statistically significant per the Log Ratio Change analysis. In contrast, the EC_50_ of linezolid decreased in four out of nine co-culture experiments and none of the changes were statistically significant.

### 2.2. Host Defense Peptide Assay

**Figure 3:**
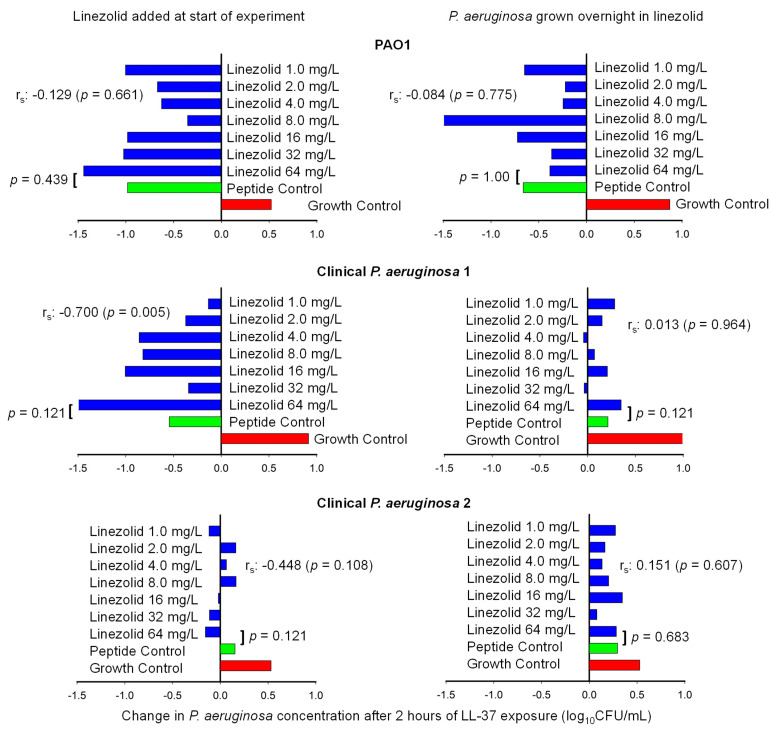
The change in the *P. aeruginosa* concentration is depicted after two hours of incubation without peptide exposure (red), exposure to LL-37 peptides (green), or exposure to LL-37 peptides and linezolid (blue). Panels on the left correspond to experiments in which linezolid and LL-37 were both added to the experiments simultaneously, whereas panels on the right depict *P. aeruginosa* that was grown overnight in linezolid prior to LL-37 exposure. The *P. aeruginosa* concentration after 2 h of exposure to LL-37 alone or LL-37 and 64 mg/L of linezolid were compared using a Mann–Whitney U Test, and the association between linezolid concentration and *P. aeruginosa* survival was determined by calculating Spearman’s correlation coefficient (r_s_).

After assessing the ability of linezolid to kill *S. aureus* through ribosomal inhibition in time-killing experiments, we then assessed the ability of linezolid to combat *P. aeruginosa* through immune activation. The ability of linezolid to potentiate the activity of the host defense peptide LL-37 is summarized in Figure 3. When the antipseudomonal activity of the peptide without linezolid was compared to the activity of the peptide during the presence of 64 mg/L of linezolid, there were no statistical differences observed for any of the three *P. aeruginosa* isolates (Mann–Whitney U Test, *p* > 0.120). Similarly, there was no correlation between the survival of PAO1 or the second clinical *P. aeruginosa* isolate and the concentration of linezolid (*p* ≥ 0.108). Exposing *P. aeruginosa* to LL-37 and linezolid simultaneously and growing *P. aeruginosa* overnight in linezolid did not greatly impact the results for PAO1 and the second clinical *P. aeruginosa* isolate.

For the first clinical *P. aeruginosa* isolate, the outcome of the experiments did vary based on the timing of the linezolid. When linezolid and LL-37 were added to the experiment simultaneously, there was a negative correlation between the concentration of linezolid and the survival of *P. aeruginosa* (r_s_ = −0.700, *p* = 0.005). When the same isolate was grown overnight in linezolid, there was not a significant association between the linezolid concentration and the amount of *P.* aeruginosa (*p* = 0.964).

## 3. Discussion

During the treatment of wound infections, there are a limited number of antistaphylococcal agents available both orally and intravenously. Several β-lactam antimicrobials such as penicillinase-resistant penicillins and first-generation cephalosporins are commonly directed against methicillin-susceptible *S. aureus* (MSSA), but such agents have restricted use in patients with β-lactam allergies or infections caused by methicillin-resistant *S. aureus* (MRSA). The protein synthesis inhibitors doxycycline, linezolid, and clindamycin may all be delivered via multiple routes of administration and are treatment options for both MSSA and MRSA [16,29]. Depending on the *S. aureus* strain, linezolid may retain activity against isolates that are resistant to clindamycin and doxycycline [30,31,32], but the use of linezolid must be balanced against the cost and adverse effects associated with the drug [16,33]. Prior studies have observed that *P. aeruginosa* is likely capable of protecting *S. aureus* from exposure to tetracyclines [6,10] and clindamycin [14], leaving linezolid as one of the few protein synthesis inhibitors with questionable utility against mixed cultures of *S. aureus* and *P. aeruginosa*.

Here, we assessed the ability of linezolid to kill *S. aureus* during an in vitro co-culture with *P. aeruginosa*, and we also evaluated if linezolid is capable of activating one component of the immune system (LL-37) to better kill *P. aeruginosa*. Relative to the comparator drug clindamycin, the presence of *P. aeruginosa* did not have a consistent impact on the maximal antistaphylococcal activity of linezolid, and the only significant changes observed were a reduction in the E_max_ in 2/9 co-culture experiments. Similarly, the potency of linezolid was not appreciably altered by *P. aeruginosa*.

The ability of linezolid to combat skin and soft tissue infections was previously assessed in a randomized trial that compared the use of linezolid to aminopenicillin–beta-lactamase inhibitor products for the treatment of foot infections in patients living with diabetes [18]. The clinical cure rate for patients infected with both Gram-positive and Gram-negative pathogens was 80% when linezolid was used in combination with aztreonam in comparison to 80% when linezolid was used alone. Patients that cultured a *Pseudomonas* species from their infection experienced an 81% cure rate in the linezolid arm versus 64% in the aminopenicillin–beta-lactamase inhibitor group. Taken together, the results of the trial may suggest that eradicating Gram-positive bacteria is the most critical aspect of treating a polymicrobial wound infection, but one competing hypothesis may be that linezolid is capable of sensitizing some Gram-negative bacteria to clearance by the immune system. Previous studies have demonstrated that certain antimicrobials that are individually not active against a pathogen may still be capable of enhancing the killing of bacteria by host defense peptides [27,28]. In the present study, there was only one experiment in which an association between the linezolid concentration and the killing of *P. aeruginosa* by the host defense peptide LL-37 was observed, suggesting linezolid does not appreciably increase the LL-37-mediated clearance of *P. aeruginosa*.

A surprising result of the current investigation was the opposing effects that *P. aeruginosa* exerted on the antistaphylococcal activity of the comparator drug clindamycin. Prior studies have observed that *P. aeruginosa* may alter the activity of antistaphylococcal agents through several mechanisms, including the release of extracellular lytic enzymes and phenotypic changes in *S. aureus* induced by exposure to small molecules such as 4-hydroxyquinoline *N*-oxide [5,34,35]. Radlinksi, et al. previously observed that the modulation of the antistaphylococcal activity of antibacterial drugs is likely multifactorial, and based on the exoproducts of a given *P. aeruginosa* strain, the impact on the drug may vary. In the current investigation, we observed two consistent but opposing phenomena in the co-culture experiments, which were the decrease in the maximal antistaphylococcal activity of clindamycin and an increase in the potency of the drug [5]. A pharmacokinetic study of clindamycin observed a C_max_ concentration of approximately 14.95 mg/L in healthy volunteers following a 900 mg IV dose, and clindamycin displayed saturable non-linear protein binding [36]. The EC_50_ concentrations of clindamycin in the current investigation were less than 0.3 mg/L in all the experiments, with concentrations of 1 mg/L approaching the E_max_ of the *S. aureus* isolates. It is, therefore, likely that the decrease in the maximal killing of clindamycin may be the more relevant observation clinically considering the ease with which maximal killing may be achieved at clinical concentrations of the drug.

The current study has several noteworthy limitations that should be considered when interpreting the results of the investigation. First, all the experiments used planktonic conditions with free-floating bacterial cells. In a wound infection, the biologic matrix of skin and soft tissue and possibly biofilms produced by the bacteria may impact the pharmacodynamics of the antibacterial agents. Second, the concentrations of linezolid used in the time-killing experiments were static. Future experiments with dynamic models will allow for the better characterization of the comparative pharmacodynamics of different antimicrobial regimens used to combat *S. aureus* and *P. aeruginosa* co-cultures. Third, bovine plasma was used when COL was cultured with PAO1, and comparisons between experiments that used bovine plasma and experiments without bovine plasma should be performed with caution. Lastly, the use of in vitro time-killing experiments is not a perfect simulation of the much more complex in vivo environment.

## 4. Materials and Methods

### 4.1. Bacterial Isolates

Three isolates of *S. aureus* and three isolates of *P. aeruginosa* were used in this study. Two separate isolates duos of *S. aureus*. and *P. aeruginosa* were collected from the polymicrobial wound infections of two separate patients treated at San Joaquin General Hospital, French Camp, CA, USA. PAO1 was used as a reference strain for *P. aeruginosa*, and COL was used as a reference strain for *S. aureus*. All three of the *S. aureus* isolates possessed a linezolid MIC of 2 mg/L, whereas the clindamycin MIC was 0.125 mg/L for COL and 0.125–0.25 mg/L for the two clinical *S. aureus* isolates.

### 4.2. Time-Killing Experiments

Time-killing experiments were conducted in 50 mL conical tubes using cation-adjusted Brain Heart Infusion (BHI) broth as described previously [14]. In brief, 10^6^ CFU/mL of each *S. aureus* isolate was investigated in time-killing experiments alone and then during separate co-culture experiments with 10^6^ CFU/mL of one of the three *P. aeruginosa* isolates, which resulted in 12 experimental iterations (three monoculture and nine co-culture experiments). An initial 20 mL suspension of bacteria was exposed to either a concentration array of clindamycin or linezolid (AK Scientific, Union City, CA, USA) that ranged from 0–64 mg/L, and 100 mcl samples were collected at 0, 2, 4, 6, 8, and 24 h, serially diluted, and plated onto BHI agar imbued with 8 mg/L of polymyxin B. After 24 h of growth, colonies were enumerated to quantify the amount of *S. aureus*. All experiments were replicated at least once.

Bovine plasma may be used in time-killing experiments to create a matrix more similar to a wound infection and potentially shield *S. aureus* from *P. aeruginosa* exoproducts [10]. In the preliminary experiments, the presence of bovine plasma interfered with the growth of both clinical *S. aureus* isolates. We also observed that COL was capable of growing unabated without bovine plasma when cultured with either of the clinical *P. aeruginosa* isolates. Bovine plasma was, therefore, only used for experiments that used the two reference strains COL and PAO1, as previously described [14].

### 4.3. Time-Killing Data Analysis

To determine if the amount of *S. aureus* killing achieved after 24 h of drug exposure was altered by the presence of *P. aeruginosa*, the Log Ratio Change was used as described in the literature [37]. In short, the concentration of bacteria at the start of the experiment and at 24 h were compared according to Equation (1).

(1) $$Log\;Ratio\;Change={Log}_{10}(\frac{{CFU}_{24}}{{CFU}_{0}})$$

The relationship between the drug concentration and the Log Ratio Change was then described using a Hill-type mathematical model (Equation (2)), where E_max_ represents the maximal killing of linezolid or clindamycin and the EC_50_ is the concentration of the drug needed to achieve half of the maximal killing (SigmaPlot 15.1.1.26). If the standard error of either the E_max_ or EC_50_ were greater than 50%, then the sigmoidicity constant H was fixed to five. The resulting parameter estimates and standard errors were used to construct 95% confidence intervals for the E_max_ and EC_50._ For each experiment, the Log Ratio Change of the growth control was subtracted from the Log Ratio Change of the other experimental arms to normalize the data. Model-generated uncertainties were used to calculate 95% confidence intervals for each parameter estimate, with non-overlapping confidence intervals indicating significantly different parameter estimates for monoculture experiments and co-culture comparisons.

(2) $$E={\;E}_{0}-\frac{E_{\max}\;\times \;{\left(C\right)}^{H}}{{\left({\mathrm{EC}}_{50}\right)}^{H}+({C)}^{H}}$$

### 4.4. Host Defense Peptide Assay

An LL-37 assay was used to help determine if linezolid will sensitize *P. aeruginosa* to killing by host defense peptides as reported by other researchers [38]. Using 15 mL conical tubes, a 5 mL suspension was created that consisted of cation-adjusted Mueller–Hinton Broth, RPMI 1640 medium, LL-37 peptides (Peptide Sciences, Batch 500297), *P. aeruginosa*, and 0–64 mg/L of linezolid. *P. aeruginosa* was either exposed to linezolid and LL-37 simultaneously or, in separate experiments, the *P. aeruginosa* was grown overnight in linezolid.

The starting inoculum, duration of LL-37 exposure, and concentration of the peptide used in the experiments were modulated in preliminary experiments to find conditions that prevented the logarithmic growth of the *P. aeruginosa* without eradicating the bacteria. After exposing 10^5^ CFU/mL of *P. aeruginosa* to 25 mg/L of LL-37 for two hours, the resulting concentration of *P. aeruginosa* remained within 1 log_10_CFU/mL of the starting inoculum. Future experiments adopted the same design and used a 10^5^ CFU/mL inoculum of *P. aeruginosa*, an LL-37 concentration of 25 mg/L, and the experiments were conducted over 2 h.

To quantify the change in the *P. aeruginosa* concentration, 100 mcl samples were collected after 0, 1, and 2 h of exposure to the LL-37, serially diluted, and plated onto Mueller–Hinton Agar. The median survival of *P. aeruginosa* observed after duplicated runs was compared for 64 mg/L and 0 mg/L of linezolid exposure using a Mann–Whitney U test (IBM SPSS Statistics Version 28) The association between the linezolid concentration and *P. aeruginosa* survival was determined by calculating Spearman’s correlation coefficient (r_s_).

## 5. Conclusions

In summary, the antistaphylococcal activity of linezolid was only minimally impacted by the presence of *P. aeruginosa* in comparison to clindamycin during the experimental conditions used in the current manuscript. Linezolid was also unable to demonstrate a consistent enhancement of *P. aeruginosa* killing by the host defense peptide LL-37. These data suggest that linezolid may be a viable antistaphylococcal agent in wound infections caused by *S. aureus* and *P. aeruginosa*, but further investigations are needed to fully characterize the optimal antibacterial regimen for polymicrobial wound infections.

## Figures and Tables

**Table 1:** A numerical summary of the parameter estimates obtained from the Log Ratio Change analysis of the time-killing data is presented. PAO1 and COL are common laboratory strains of *P. aeruginosa* and *S. aureus*, respectively, that were used as references. Clinical *P. aeruginosa* and *S. aureus* isolates were collected from polymicrobial wound infections. The maximal killing (E_max_) and potency (EC_50_) of linezolid and clindamycin are listed along with 95% confidence intervals listed parenthetically. Confidence intervals of parameter estimates generated from polymicrobial experiments that did not overlap with confidence intervals of corresponding monoculture experiments are indicated with an asterisk (*). The adjusted R^2^ was >0.9 for all the Hill-functions used to describe the time-killing data.

*S. aureus* Isolate	*P. aeruginosa* Isolate and Number of Experimental Replicates (Linezolid and Clindamycin)	Linezolid E_max_ (95% CI)	Linezolid EC_50_ (95% CI)	Clindamycin E_max_ (95% CI)	Clindamycin EC_50_ (95% CI)
COL (plasma)	None (2, 2)	3.87 (3.42–4.32)	1.37 (1.04–1.70)	6.09 (5.39–6.78)	0.17 (0.12–0.22)
	PAO1 (2, 2)	4.24 (3.92–4.55)	1.91 (1.49–2.33)	4.66 (3.72–5.60)	0.093 (0.025–0.16)
COL	None (2, 2)	5.22 (4.84–5.61)	1.81 (1.42–2.21)	5.84 (5.34–6.34)	0.13 (0.076–0.18)
	Clinical 1 (2, 2)	4.12 (3.60–4.77) *	1.75 (1.03–2.46)	4.51 (4.28–4.74) *	0.066 (0.059–0.072) *
	Clinical 2 (2, 2)	4.04 (3.68–4.40) *	1.87 (1.37–2.36)	4.30 (3.93–4.66) *	0.069 (0.056–0.081)
Clinical *S. aureus* 1	None (2, 2)	4.79 (4.03–5.54)	2.96 (2.07–3.85)	6.37 (5.37–7.38)	0.22 (0.17–0.27)
	PAO1 (2, 2)	4.77 (3.89–5.65)	2.82 (1.27–4.37)	4.58 (3.50–5.65)	0.051 (0.022–0.079) *
	Clinical 1 (2, 3)	3.89 (3.35–4.43)	3.28 (2.04–4.51)	4.24 (3.63–4.86) *	0.11 (0.060–0.16) *
	Clinical 2 (2, 2)	4.03 (3.02–5.04)	3.07 (0.91–5.23)	3.92 (3.40–4.44) *	0.065 (0.044–0.086) *
Clinical *S. aureus* 2	None (3, 2)	4.24 (3.48–5.00)	3.19 (−1.77–8.14)	5.56 (4.30–6.83)	0.20 (0.13–0.27)
	PAO1 (4, 2)	4.48 (−0.22–9.18)	2.29 (−8.18–12.76)	5.30 (3.91–6.69)	0.078 (0.0068–0.15)
	Clinical 1 (5, 2)	4.39 (3.39–5.39)	2.62 (0.70–4.53)	5.81 (4.07–7.54)	0.17 (−0.012–0.35)
	Clinical 2 (3, 2)	4.31 (3.31–5.31)	3.23 (0.84–5.62)	4.10 (3.49–4.71)	0.084 (0.041–0.13)

## Data Availability

Data available on request.

## Abbreviations

The following abbreviations are used in this manuscript:

MRSA	Methicillin-resistant *S. aureus*
MSSA	Methicillin-susceptible *S. aureus*
